# Substance use among students of two high schools in Yaoundé, Cameroon

**DOI:** 10.11604/pamj.2024.49.121.43487

**Published:** 2024-12-16

**Authors:** Hilda Tonge Ekwoge, Sonita Anjei Mbah, Joseph Nelson Siewe Fodjo, Germaine Likowo Mbange, Leonard Ngarka, Felicien Enyime Ntone, Alfred Kongnyu Njamnshi

**Affiliations:** 1 HILPharma Association, Yaoundé, Cameroon,; 2 Internal Medicine Department, Faculty of Medicine and Biomedical Sciences, University of Yaoundé I, Yaoundé, Cameroon,; 3 Global Health Institute, University of Antwerp, Antwerp, Belgium,; 4 Brain Research Africa Initiative (BRAIN), Yaoundé, Cameroon,; 5 Psychiatry Service, University Teaching Hospital, Yaoundé, Cameroon

**Keywords:** Substance use, drugs, risk factors, schools, Yaoundé, Cameroon

## Abstract

**Introduction:**

substance use among adolescents is on the rise, estimated at 15.3% in Buea, Cameroon (2021). However, factors associated with this practice are still understudied in the Cameroonian setting.

**Methods:**

a cross-sectional study (January to May 2022) was conducted to document substance use among students of two high schools in Yaoundé, Cameroon. Socio-demographics, psychosocial parameters, and history of substance use were documented among students via self-administered questionnaires. Validated tools (including the PHQ-4 and the 7-item Weinberger adjustment inventory) were used to assess anxiety, depression, self-esteem, and other youth behavioural risks. Descriptive analyses and multiple logistic regression were performed.

**Results:**

we recruited 373 students (43.0% male, mean age: 16 ± 1.6 years). Overall, 42.4% had already consumed at least one substance, notably alcohol (40.5%), vap/shisha (8.3%), and tobacco/cigarettes (2.7%). Previous tramadol use was reported by 6 (1.6%) participants. The multiple logistic regression analysis found that the following factors were associated with increased odds of lifetime substance use among students: male gender (OR: 2.013, 95% CI: 1.127-3.595), depression (OR: 1.238, 95% CI: 1.031-1.487) and low self-esteem (OR: 1.083, 95% CI: 1.003-1.168). Meanwhile, increased parental concerns about drug use served as a protective factor for substance use among the students (OR: 0.196, 95% CI: 0.078-0.496).

**Conclusion:**

about two-fifths of the enrolled high school students had already consumed one or more psychoactive substances, highlighting the need for urgent preventive interventions. For optimal impact, substance use preventive programs should include self-esteem building among the students and improved parental attitudes towards drugs.

## Introduction

Drug and substance abuse is a major health problem worldwide. According to the World Drug Report 2023, an estimated 296 million people aged 15-64 years used drugs worldwide in 2021, representing a 23% increase over the previous decade [1]. Increased consumption of alcohol, tobacco, and marijuana (cannabis) has also been observed, particularly during the COVID-19-induced lockdowns [2]. Besides the health issues caused by substances like alcohol and tobacco, their consumption also engenders significant economic costs [3].

Most research suggests that adolescence (12-17 years old) is a critical risk period for the initiation of psychoactive substance use, especially marijuana (cannabis), alcohol, and tobacco [2]. Remarkably, substance use is increasingly frequent among the youth; indeed, global reports show that marijuana use is more frequent among the 15-16-year-olds compared to the rest of the population [1,4]. As for alcohol, it is the most commonly consumed substance with 26.5% of all 15-19-year-olds reported to be current drinkers (corresponding to about 155 million adolescents worldwide) [5]. The proportion of adolescent alcohol consumers in sub-Saharan Africa (SSA), estimated at 32.8% [6], is slightly higher than the worldwide prevalence of alcohol consumption. Tobacco, on the other hand, has already been consumed by one in four adolescents in SSA [6]. Another addictive substance that is commonly used by SSA youths is tramadol (an opioid agonist available in the form of prescription tablets) [7,8]. The concerns over substance use are increasingly drawing the attention of the international community toward the significant risk of drug overdose death, particularly in adolescents [9].

In Cameroon, over 12,000 children below 16 years of age had reportedly used narcotic and psychotropic substances [10]. Already in 1996, it was documented in Cameroon that up to 20% of drug users started experimenting with psychoactive substances before the age of 15 years [11]. Drug consumption within the school milieu is taking alarming proportions, with devastating side effects on both the consumers and the school milieu (increased violence, juvenile delinquency, etc.) [12]. A study carried out by the Global Youth Tobacco Survey (GYTS) in Cameroon in 2008 found that 44% of young students had already used drugs at least once [13]. More recent studies in Cameroon also confirm substance use among both high school students [14] and university students [10,15,16]. While the phenomenon of substance use appears to be on the rise, there still exist knowledge gaps regarding substance use among adolescents in Cameroon, and the risk factors involved.

Understanding the individual and environmental factors associated with substance among the youth in the Cameroonian setting is crucial for the development and implementation of context-relevant health policies. Cameroon currently subscribes to both national and international statutes which regulate the sales of narcotic and psychotropic substances, and prohibit their misuse/abuse [17]. Furthermore, the government has developed policies specifically addressing alcohol use (in the community and workplaces, drink-driving, availability, marketing, and pricing) [18] as well as tobacco use (prohibiting cigarette smoking and banning water pipes/shisha) [18,19], but they are often poorly implemented resulting in little change in practices.

In this study, we sought to investigate the frequency and extent of substance use among students from two high schools in Yaoundé (Cameroon) and highlight the circumstances surrounding this phenomenon in our own setting. The public health implications of our findings reside in their usefulness for implementing effective interventions that would curb the upward trends in juvenile substance use and prevent adverse health outcomes in this population.

## Methods

**Study design and setting:** we conducted a cross-sectional study between January and May 2022 in two public high schools in Yaoundé, Cameroon. Yaoundé is the political capital of Cameroon with a population of over 2.5 million. It lies in the central region of the nation at 750 m above sea level. The two public schools where the study was conducted were: Government Bilingual High School Etoug-Ebe (also known as “Lycée Bilingue d´Etoug-Ebe”, abbreviated LBE), and Government Bilingual Practising High School Yaoundé (also known as “Lycée Bilingue d´Application”, abbreviated LBA). These two schools were chosen by convenience in view of their large student population (over 4000 registered students per school) and anecdotal reports of substance use by some of their students.

**Study procedures:** we started by meeting with the school authorities to discuss the study with them and obtain administrative permission to conduct the research. To avoid distracting students who were preparing for national examinations, the school administration granted us permission to approach only the students attending the classes of form 3, form 4, and lower sixth for inclusion in the study. The students were encountered in their classrooms, in the presence of their teachers, who stood in as their guardians. A 5-minute mini-lecture was given to explain the study and identify students who agreed to participate. All assenting students aged 12 years old and above who were willing and available at the time of study were included. Students who verbally acknowledged suffering from a brain/mental condition such as schizophrenia, epilepsy, and others were excluded. The research team encountered each of the selected classrooms, and the students were invited to participate one after the other following a consecutive recruitment strategy. Only the English-speaking sections of the participating schools were involved in the study to ease the administration of the questionnaire in English.

**Data collection:** the research team provided instructions about how to fill the data collection tool; thereafter, a blank questionnaire was handed to each student who consented to participate. At least one research team member was present in the classroom during the filling of the self-administered questionnaires to facilitate the process and offer guidance to the participants. The data collection instrument was developed by reviewing previous studies that addressed the topic of substance use and associated risk factors [20]. The following data were collected: socio-demographic information; sexual activity of the participant; school performance indicators; psychosocial well-being (anxiety and depression screening); social environment of participants in their households/communities; and substance use by the participants and their parents. Validated tools were used to assess various psychosocial parameters in the participants, with minor contextual adaptations when needed (Table 1).

**Table 1:** summary of psychosocial tools in the study questionnaire

Parameter to measure	Name of tool used	Brief description	Interpretation of score
Anxiety and depression	Patient Health Questionnaire (PHQ-4)^a^	Four multiple choice questions: The first two for anxiety, and the last two for depression. Overall range: 0 - 12 (0 - 6 for anxiety, and 0 - 6 for depression)	A higher score implies more anxious or depressive tendencies; a score ≥3 for each parameter (anxiety and depression) is considered a positive screening
Self-esteem	7-item Weinberger adjustment inventory^b^	Measures an individual’s perception of his or her own value. Each item is scored between 1 and 5 (Overall range: 7 - 35)	The higher the score, the lower the self-esteem
Parental control	Houston community demonstration project for Behaviour assessments^c^ (modified)	Measures the amount and kind of television programs parents allow their children to watch. It also measures the extent to which parents know their children’s friends and tastes; range: 1 - 4 for each item	Point values were summed and divided by the number of responses. A higher mean score implies high parental control, and a lower mean score indicates low parental involvement
Parental attitudes toward drug use	Seattle social development project^d^	Measures the youth’s perception of their parents’ attitudes about drinking and taking drugs; range: 1 - 4 for each item	Point values were summed and divided by the number of responses. A higher score indicates greater parental concern about drug use
Family hostility	Rochester youth development study^e^	Measures the extent to which parents report a climate of hostility and conflict within the family; range: 1 - 4 for each item	Point values were summed and divided by the number of responses. A higher score indicates a higher level of hostility and conflict within the family
Drugs and alcohol use	Youth risk behaviour and teen conflict survey^f^ (modified)	Measures the frequency of drug use in the past months, as well as the recent episodes of substance use; range: 0 - 24	Scores were derived by summing all responses. A higher score indicates high drug/alcohol activity

a Kroenke K *et al*. Psychosomatics. 2009;50(6):613-621; ^b^ Weinberger DA *et al*. Weinberger Adjustment Inventory. 2012. doi:10.1037/t05237-000; ^c^ Dahlberg LL *et al*. Houston Community Demonstration Project. 1993; ^d^ Dahlberg LL *et al*. Parental Attitudes Toward Drug Use - Seattle Social Development Project. 2005; ^e^ Krohn MD *et al*. Rochester Youth Development Study and Rochester Intergenerational Study. The Encyclopedia of Research Methods in Criminology and Criminal Justice. 2021. Wiley: 117-125; ^f^ Underwood JM. Overview and Methods for the Youth Risk Behavior Surveillance System - United States, 2019. MMWR Suppl. 2020;69. doi:10.15585/mmwr.su6901a1

Data were collected for six psychosocial parameters; three of these parameters related to the participant´s state (anxiety, depression, and self-esteem), while the three others related to the participant´s environment (parental control, parental attitude towards drug use, and handling misunderstandings at home). Guidelines for the proper interpretation of these psychosocial parameters are summarised in Table 1.

**Definition of terms:** substance: in this article, a substance is defined as any psychoactive compound with the potential to cause health and social problems, including addiction [21]. This generally includes ten separate classes of drugs: alcohol, cannabis, hallucinogens, inhalants, opioids, sedatives, hypnotics (or anxiolytics), stimulants (including amphetamine-type substances, cocaine, and other stimulants), and tobacco [22]. Substance use: refers to the intake of any psychoactive substance for whatsoever reason, irrespective of the route of administration [23]. Substance misuse: implies the intake of psychoactive substances at high doses or in inappropriate situations that could cause a health or social problem. When this situation becomes prolonged and repeated, it could become a substance use disorder (also known as substance abuse). Severe and chronic substance use disorders are commonly called addictions [21].

**Sample size calculation:** Cochran´s formula for calculating sample size in cross-sectional studies was used in this study, as follows:


$$n=\frac{z^{2}p\left(1-p\right)}{d^{2}}$$

Where: n = sample size; Z = standard normal variant at a confidence interval of 95% (standard value 1.96); P = expected proportion in population; d = margin of error (0.05). Assuming that the frequency of drug use in the different sites is equal to the national prevalence of 10% [13], at least 139 participants were required from each study site.

**Data management and analysis:** all data collected were entered into Microsoft Excel 2016 and exported to the software R version 4.2.2 for analysis. Categorical variables were presented as frequencies and proportions and compared across groups using the Chi-square test (or Fisher test where appropriate). Continuous variables were presented as means (and standard deviation) and compared across groups using the Mann-Whitney U test. Besides descriptive analysis, we conducted multiple logistic regression analyses to identify factors associated with substance use among the students. For the multivariable regression analysis, age and sex were compulsory variables together with other covariates which produced a p-value <0.2 during univariate analysis. For both descriptive and inferential statistics, missing data were excluded. A significance threshold of 5% was adopted.

**Ethical considerations:** all procedures were approved by the institutional review board (IRB) of the Faculty of Medicine and Biomedical Sciences of the University of Yaoundé I (Ref: N°32/UYI/FMSB/VDRC/CFD of 28/04/2022). Participation was totally voluntary. Administrative permissions were obtained from the school authorities, and the teachers of each participating class stood in as consenting guardians. The questionnaires were filled anonymously and analyzed with strict confidentiality.

## Results

**Description of study participants:** a total of 373 participants were recruited from the two high schools, of whom 180 (57.0%) were females (Table 2). The participating students´ ages ranged from 12-22 years, with only 8 (2.1%) of them being older than 19 years (i.e. non-adolescents). Significant differences existed between schools, notably regarding the participants´ age and academic performance.

**Table 2:** socio-demographic characteristics of the study participants by school

	Overall findings	Total N^*^	LBA (n=133)	LBE (n=240)	P-value
Age in years: mean (SD)	16.0 (1.6)	363	15.0 (1.9)	16.6 (1.1)	<0.001
**Gender: n (%)**		**316**			**1.000**
Female	180 (57.0%)		71 (57.3%)	109 (56.8%)	
Male	136 (43.0%)		53 (42.7%)	83 (43.2%)	
**School level: n (%)**		**373**			**<0.001**
Form 3	39 (10.5%)		39 (29.3%)	0 (0%)	
Form 4	53 (14.2%)		53 (39.8%)	0 (0%)	
Lower sixth	281 (75.3%)		41 (30.8%)	240 (100%)	
**Religion: n (%)**		**342**			**0.340**
Islam	17 (5.0%)		9 (7.4%)	8 (3.6%)	
Christianity	321 (93.9%)		112 (91.8%)	209 (95.0%)	
Other	4 (1.2%)		1 (0.8%)	3 (1.4%)	
School performance (on 20) during the first term of the academic year: mean (SD)	11.0 (2.6)	213	12.5 (2.5)	10.1 (2.2)	<0.001

* N: total number of students with valid data for that variable, excluding participants with missing data; LBA: *Lycée Bilingue d’Application*; LBE: *Lycée Bilingue d’Etoug-Ebe*; SD: standard deviation

**History of lifetime substance use among the participants:** the overall prevalence of lifetime substance use in our study was 158/373 (42.4%). The most consumed substance was alcohol (40.5%), with significant differences across schools: 32.3% in LBA compared to 45.0% in LBE (p=0.023) (Table 3). The next most consumed substance was vap/shisha, consumed by 5.3% of participants in LBA and 10.0% of participants in LBE (p=0.164). Considering all the substances, a history of previous consumption was more frequent among the male participants (71/136; 52.2%), compared to the female participants (67/180; 37.2%); p=0.011.

**Table 3:** the proportion of participants with a history of consuming psychoactive substances

Lifetime substance use: n (%)	Overall findings	Total N^*^	LBA (n=133)	LBE (n=240)	P-value
Ever consumed alcohol	151 (40.5%)	373	43 (32.3%)	108 (45.0%)	0.023
Ever consumed vap/shisha	31 (8.3%)	373	7 (5.3%)	24 (10.0%)	0.164
Ever consumed tobacco	10 (2.7%)	373	1 (0.8%)	9 (3.8%)	0.104
Ever consumed tramadol	6 (1.6%)	373	0 (0%)	6 (2.5%)	0.093
Ever consumed marijuana	4 (1.1%)	373	2 (1.5%)	2 (0.8%)	0.619
Ever consumed inhalants	4 (1.1%)	373	1 (0.8%)	3 (1.3%)	1.000

* N: total number of students with valid data for that variable, excluding participants with missing data; LBA: *Lycée Bilingue d’Application*; LBE: *Lycée Bilingue d’Etoug-Ebe*; SD: standard deviation

**Figure 1:**
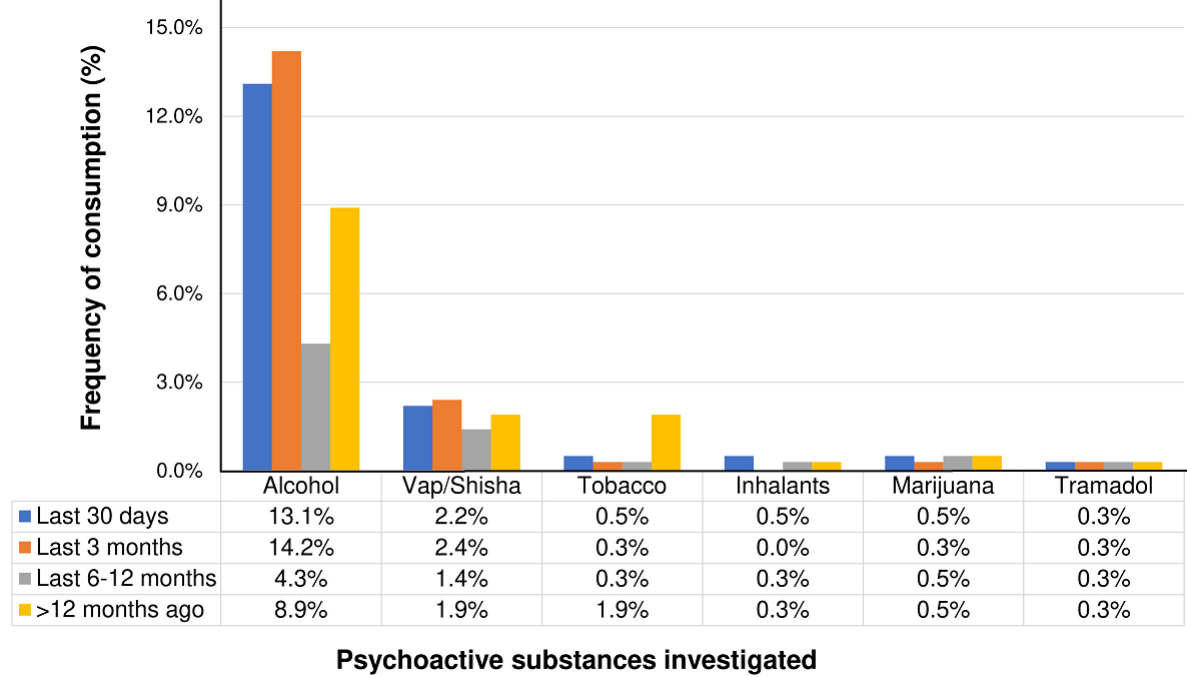
time elapsed since most recent substance use among the participants with a history of substance use (data from participants who had never consumed any substance is not shown)

**Recent substance use among students in both schools:** the elapsed time since the most recent substance use episode revealed that alcohol and vap/shisha were the most consumed substances over the past 30 days (Figure 1). Except for alcohol which had been consumed recently by a greater proportion of students from LBE (16.2% during the last 30 days vs 7.5% in LBA; p=0.045), there were no other inter-school differences in the recent substance use trends.

We also investigated the sexual activity of the participants (data available for n=359 students). Based on the available data, 50/359 (13.9%) reported to have already had at least one sexual intercourse (all heterosexual). The proportion of participants with a sexual history did not differ significantly across schools: 35/228 (15.4%) in LBE versus 15/131 (11.5%) in LBA; p=0.385. Of the 50 sexually active students, 37 (74.0%) had a history of substance use. Furthermore, the proportion of sexually active students among those with a history of substance use (37/153, 24.2%) was higher compared to that among non-users (13/206, 6.3%); p<0.001. Overall, the reported age at first intercourse ranged from 10 years to 18 years.

**Psychosocial findings among participants:** when compared across schools, none of the psychosocial outcomes differed significantly. However, participants with a history of substance use had worse psychosocial findings compared to their peers who had never taken any substance (Table 4).

**Table 4:** psychosocial findings related to substance use among study participants

Psychosocial parameters	Overall findings	Total N^*^	Participants with past history of substance use	Participants with no history of substance use	P-value#
Positive anxiety screening: n (%)	107 (30.2%)	354	57/148 (38.5%)	50/206 (24.3%)	0.006
Positive depression screening n (%)	117 (33.6%)	348	63/143 (44.1%)	54/205 (26.3%)	0.001
Low self-esteem score: mean (SD)	20.3 (4.4)	357	20.9 (4.3)	20.0 (4.5)	0.044
Parental control score: mean (SD)	3.0 (0.5)	367	2.9 (0.5)	3.0 (0.6)	0.037
Parental attitude score: mean (SD)	3.8 (0.5)	366	3.6 (0.5)	3.9 (0.4)	<0.001
Family hostility score: mean (SD)	3.1 (0.7)	361	3.0 (0.8)	3.2 (0.7)	0.006

* N: total Number of students with valid data for that variable; SD: standard deviation; #P-value for the comparison of findings among participants with and without a history of substance use, using the Chi-squared test for categorical variables, and the T-test for continuous variables

**Factors associated with substance use:** the multiple logistic regression model revealed that being a male, having a higher depression score, a higher score for low self-esteem, and a lower parental attitude score were all significantly associated with increased odds of having consumed at least one of the aforementioned substances among the participating students (Table 5).

**Table 5:** multiple logistic regression model investigating factors associated with a history of substance use (n = 277 participants with complete data)

Model^*^ covariates	Adjusted OR (95% CI)	P-value
Age in years^**^	1.157 (0.914 - 1.466)	0.226
**Gender**		
Female gender	Reference	
Male gender	2.013 (1.127 - 3.595)	0.018
**School**		
LBA	Reference	
LBE	1.192 (0.514 - 2.766)	0.682
**School level**		
Form 3	Reference	
Form 4	1.573 (0.501 - 4.939)	0.438
Lower sixth	1.365 (0.356 - 5.224)	0.650
**Sexually active**		
No	Reference	
Yes	1.892 (0.805 - 4.443)	0.143
Anxiety score^**^	1.066 (0.887 - 1.283)	0.495
Depression score^**^	1.238 (1.031 - 1.487)	0.022
Parental control score^**^	0.899 (0.514 - 1.574)	0.709
Parental attitude score^**^	0.196 (0.078 - 0.496)	<0.001
Family hostility score^**^	0.725 (0.479 - 1.098)	0.129
Low self-esteem score^**^	1.083 (1.003 - 1.168)	0.042

* 96 participants excluded due to missing data; model AIC: 340.1; ^**^continuous variable; LBA: *Lycée Bilingue d’Application*; LBE: *Lycée Bilingue d’Etoug-Ebe*; OR: odds ratio; CI: confidence interval

## Discussion

Our study conducted in two public high schools in the urban setting of Yaoundé in Cameroon found that 42.4% of the 373 recruited students had already used at least one of the investigated substances. The fact that substance use was more frequent among students from LBE compared to LBA could be due to the fact that the participants from the former school were significantly older. The overall prevalence of substance use in our study is in a similar range as previous findings in South Africa, notably 47% in the Free State Province [24] and 31% in Johannesburg [25]. However, other studies found lower values such as the 17.3% in Ado-Ekiti South West of Nigeria [26], 9.6% in Bangladesh [27], and 9.3% in Morocco [28]. Meanwhile, other studies found a higher prevalence of substance use among adolescents, such as 55.9% in Kenya [29] and 52.5% in Ethiopia [30]. A previous study among 399 street children (aged 12 to 19 years) in Cameroon revealed that all of them were actively using psychoactive substances [31,32]. The discrepancies observed could be due to socio-cultural differences across the study populations. Differences in the prevalence of substance use within Cameroonian sites could also be related to the security context. In the South-West region where there is an ongoing armed conflict, some of the highest rates of substance use have been reported in recent years [33]: alcohol (62.5%), cigarette smoking (23.8%), tramadol (12.1%), marijuana (9.6%), and hard drugs (>3%).

Alcohol stood out as the most consumed substance in our study (40.5%), concurring with a previous study in which 45.9% of Cameroonian street children preferred alcohol as their main psychoactive substance [31], and another study among the Baka (South-eastern Cameroon) where 45% of male adolescents had already consumed alcohol [34]. More recently in Buea (South West Region of Cameroon), Nkouonlack *et al*. found that 32.7% of secondary school students consumed alcohol [14]. Similar proportions of alcohol consumers were observed among adolescents in Free State Province in South Africa (40.7%) [24], Botswana (42.1%) [35], and Kenya (43.4%) [29]. In Kisumu (Kenya), up to 57.9% of adolescents had already consumed alcohol [36] while in Ethiopia, a record 63.1% of high school students reported lifetime alcohol consumption [30]. In contrast, much lower alcohol consumption rates were found in Morocco (4.3%) [28] and 2.3% in Bangladesh [27] most likely because those were Muslim communities who abstain from alcohol for cultural reasons. Meanwhile, in Cameroon, alcohol consumption is deeply anchored in societal customs and has even been associated with prestige and maturity [37]. Contributing to the relative acceptability and accessibility of alcohol in Cameroonian settings are the sub-optimal policies; indeed, policy areas such as marketing alcoholic beverages, pricing, drink-driving countermeasures, and community, and workplace actions all scored under 40 points (on a scale of 0-100) upon analysis [38]. A presidential decree dated 9^th^ November 1990 specified that sales points for alcoholic beverages must not be found within 200 m of schools (among other institutions) [39] but this is seldom observed by the population. Therefore, in addition to improving and enforcing the implementation of the existing policies, practical solutions such as setting a minimum age for purchasing alcoholic beverages could also be instituted to curb the observed trends in alcohol consumption by adolescents.

Vap/shisha emerged as another upcoming substance among the youth in our study. Indeed, we found that up to 8.3% of our participants had a history of vap/shisha consumption. This is quite elevated considering the logistical requirements and costs associated with this practice (access to the pipes and consumables) in contrast with cigarettes which are readily available in shops. Although the frequency of vap/shisha use in our population is slightly higher than the 5% observed among high school learners in Johannesburg [25], it is much lower than the 31.4% among high school students in Jordan, who were mostly Muslims [40]. Tobacco/cigarette consumption was reported by 2.7% of our participants, lower than what is observed in predominantly Muslim settings like Morocco (16.1%) [28]. We surmise that Muslim communities tend to be more given to smoking and/or other substances as coping mechanisms, since alcohol is often prohibited in such settings. Notwithstanding, data on alcohol consumption from Muslim settings should be interpreted with caution, as the Muslim participants tend to under-report their drinking habits to researchers [41]. For the majority of students with a history of tobacco use in our study, the most recent tobacco use occurred more than one year ago; this supposes that these students succumbed to experimental smoking during their early adolescence. It is worth noting that high rates of cigarette smoking among adolescents were also reported in Kenya (22.1% to 34.7%) [29,36]. The reasons for these wide disparities with our own findings need to be further investigated. Although sound policies exist to address the smoking problem in Cameroon, their implementation remains weak [18]. Priority should therefore be placed on strengthening the compliance of the population, particularly adolescents, to the existing measures to prevent tobacco dependence in future generations. For instance, the prohibition of shisha use [19], if implemented properly and severe sanctions applied to defaulters, would certainly go a long way to push this substance out of the Cameroonian market.

Tramadol use had a prevalence of 1.1%, which is lower compared to another study among secondary school students in Buea, Cameroon, where tramadol use exceeded 10% [14]. Among university students, tramadol consumption was observed in 1.6% of medical/nursing students [15], and in 7.5% of students of tertiary institutions of Buea, Cameroon [10]. Studies conducted in Nigeria among high school students found that 3.8% to 12.9% of them had ever consumed tramadol [7,26]. Being a very accessible opiate, there is a high risk that youths become dependent on tramadol with immediate consequences in their schools and communities. Indeed, alcohol and tramadol consumption were reportedly related to violent fights, rape, and even coma in some Cameroonian communities [34]. Frequent checks and surprise field missions should be organized in pharmacies/drug stores/markets to ensure that properly procured tramadol is dispensed only after presenting a medical prescription, as required by law. Marijuana and inhalants were seldom consumed by our study participants, contrasting with a Moroccan study which reported that these substances were consumed by respectively 8.1% and 1.7% of students [28]. It appears that the Moroccan participants compensate for their avoidance of alcohol by consuming other psychoactive substances.

In the multiple logistic regression model, the independent predictors of substance use that emerged included: being male, being screened positively for depression, parental attitude towards substance use, and low self-esteem. This is close to previous findings from Buea, Cameroon, where the male gender, increasing age, and being a student constituted risk factors for substance use [33]. Concerning parental attitude, the inverse relationship observed with substance use implies that parents who display a greater degree of tolerance towards substances with potential for abuse are more likely to have children who will become substance use victims. Our findings align with those from a study in Botswana which showed that being male and having a parent who uses drugs increases the risk of drug use [35]. Therefore, a sustainable and impactful anti-substance abuse intervention for adolescents should inevitably include parents, guardians, and community leaders who would create a conducive social environment to consolidate the efforts done by teachers in schools [42]. The fact that parental control and family hostility scores were not significantly associated with substance use in the adjusted model requires further investigations in which the parents themselves will be involved possibly using a mixed-methods design.

The association between self-esteem and substance use has previously been documented by abundant literature, showing that lower self-esteem is associated with increased substance use among adolescents and vice versa [43,44]. In our study, a higher score on the “low self-esteem scale” implied worse self-esteem and was associated with significantly higher odds of substance use. This result underscores the importance of building self-confidence and resilience among adolescents as a means of preventing substance use in the future. Indeed, a higher self-esteem is likely to make teenagers more resilient to the peer pressure to use drugs. Such an approach has been adopted by the substance use prevention programme recently launched by HILPharma Association in Cameroon [45].

**Study limitations:** our study had some limitations. The veracity of the information provided by the students would be difficult to verify, and there is a risk of both recall and social desirability biases in the responses. Also, the sample size for the LBA high school was short by six participants; however, we overcame this limitation by joining all the study sites for the multivariable analysis to investigate the risk factors for substance use. The fact that we could not recruit from all the classrooms and also did not include French-speaking students certainly limits the generalisability of our findings, although we do not expect marked differences between French-speaking and English-speaking students within the same school. Also, the history of previous/recent substance use does not clearly indicate if the participants were addicted since the addictive component requires data on habitual substance use. Future studies could consider translating and validating the research tools in French to obtain empirical data from other students in our study sites and other schools as well. Finally, our purely quantitative study design can only give a partial image of the substance use situation in these schools, requiring a qualitative approach to complement the findings.

## Conclusion

Two in every five high school students in the surveyed high schools in Yaoundé (Cameroon) had already consumed one or more psychoactive substances. While societal habits and parental attitudes seem to contribute to this trend, the adverse consequences of such practices represent an impending danger both for the individual and society at large. Although these findings are not generalizable to the entire Cameroonian population, they do provide arguments for a more comprehensive approach to substance use prevention among students. Notably, interventions geared at curbing the consumption of harmful substances in schools should include the students, parents, and communities for a sustainable impact. National policies should be reinforced to restrict access to psychoactive substances by adolescents. With SSA currently witnessing a rise in substance abuse, urgent action is needed in Cameroon to fight this societal ill.

### What is known about this topic

- Substance use is on the rise among Cameroonian youth;
- Risk factors for substance vary depending on the setting and the population.

### What this study adds

- Our findings reveal that 42.4% had already consumed at least one substance, notably alcohol (40.5%), vap/shisha (8.3%), and tobacco/cigarettes (2.7%);
- Our study documents the following risk factors for substance use among students in Yaoundé (Cameroon), which could be utilized for interventional purposes: male gender, depression, low self-esteem, and low parental concern for the child.
